# Assessment of left heart dysfunction to predict doxorubicin cardiotoxicity in children with lymphoma

**DOI:** 10.3389/fped.2023.1163664

**Published:** 2023-05-05

**Authors:** Jiaqi Chen, Chunyue Cheng, Li Fan, Xiaochuan Xu, Jing Chen, Yang Feng, Yi Tang, Chunjiang Yang

**Affiliations:** ^1^ Department of Ultrasound, Children’s Hospital of Chongqing Medical University, National Clinical Research Center for Child Health and Disorders, Ministry of Education Key Laboratory of Child Development and Disorders, Chongqing, China; ^2^ Department of Ultrasound, Wuhan Children’s Hospital (Wuhan Maternal and Child Healthcare Hospital), Tongji Medical College, Huazhong University of Science and Technology, Wuhan, China

**Keywords:** speckle-tracking echocardiography, cancer treatment-related cardiac dysfunction, longitudinal strain, children, left atrial strain

## Abstract

**Objectives:**

The objectives of this study were to assess the changes in the left myocardial function after chemotherapy for childhood lymphoma and observe the predictive or monitor value for cancer treatment-related cardiac dysfunction (CTRCD) by speckle-tracking echocardiography.

**Methods:**

A total of 23 children with histopathological diagnoses of lymphoma were included, with age-matched normal controls. Comparative analysis of clinical serological tests and left heart strain parameters in children with lymphoma, including left ventricular global longitudinal strain (LVGLS); global myocardial work (GMW) indices, which include global work index (GWI), global constructive work (GCW), global wasted work, and global work efficiency; and the LS of subendocardial, middle, and subepicardial layer myocardium during left ventricular systole were measured: left atrial strain of reservoir phase (LASr), left atrial strain of conduit phase (LAScd), and left atrial strain of contraction phase (LASct).

**Results:**

One-way ANOVA showed that GLS, GWI, GCW, LASr, and LAScd were closely associated with CTRCD and multivariate logistic regression analysis showed that GLS was the most sensitive predictor for detecting patients at lofty risk of anthracycline-related cardiotoxicity. Both before and after chemotherapy, GLS in the left ventricle showed a pattern of basal segment < middle segment < apical segment and subepicardial < middle < subendocardial layer (*p* < 0.05), and the degree of decrease also showed a regular pattern of epicardial layer < middle layer < subendocardial layer while the difference was not significant (*p* > 0.05). After chemotherapy, maximum flow rate in early mitral relaxation/left atrial systolic maximum flow rate (E/A) and left atrial volume index of each group were in the normal range, and the values of LASr, LAScd, and LASct slightly increased in the second cycle and decreased significantly in the fourth cycle after chemotherapy, reaching the lowest level; LASr and LAScd were positively correlated with GLS.

**Conclusion:**

LVGLS is a more sensitive and earlier indicator to predict CTRCD compared with conventional echocardiography-related parameters and serological markers, and GLS of each myocardial layer showed a certain regularity. Left atrial strain can be used for early monitoring of cardiotoxicity in children with lymphoma after chemotherapy.

## Introduction

Anthracyclines (ANTs) are first-line drugs for lymphoma chemotherapy, and antitumor mechanisms primarily include the inhibition of DNA and RNA synthesis in cells and inhibition of topoisomerase II activity, which are toxic to cardiomyocytes while inhibiting tumor cell growth and being progressive, irreversible, and dose-dependent (1). Chemotherapy-related heart failure is an important cause of morbidity and mortality in cancer patients (2). At the same time, patients treated with ANTs, have an octuple higher risk of death caused by heart failure (3). Cancer treatment-related cardiac dysfunction (CTRCD) was defined as asymptomatic left ventricular ejection fraction (LVEF) decrease of more than 10%, or LVEF lower than normal (LVEF < 53%) after antitumor treatment, or regardless of the baseline value when the patient has clinical symptoms with more than 5% decrease in LVEF (4). LVEF was insufficient to identify the early changes of left ventricle (LV) function, whereas subclinical alterations in left ventricular longitudinal function could be detected early in the course of the disease by global longitudinal strain (GLS) parameters measured by speckle-tracking echocardiography (STE) (5–7). Except for GLS, the layered strain technique compensates for the various effects of global strain on the structure and direction of each layer of myocardium. It demonstrates the longitudinal strain damage of each layer of myocardium by reflecting the relationship between muscle fiber structure and damage of the three layers of the ventricular myocardium (8). Thus, the prominent advantage of the myocardial work technique is that considering the myocardial deformation and afterload can better reflect the myocardial energy metabolism (9–11). In addition, the left atrium (LA) is the intersection of the pulmonary and systemic circulation, which can regulate LV loading and better represent LV diastolic function; in light of experts, ventricular diastolic dysfunction frequently manifests before systolic impairment (12), and left ventricular diastolic dysfunction (LVDD) is earlier and more closely associated with the left atrium than with the left ventricular chamber (13).

We quantitatively evaluated the functional changes of LV and LA after ANT chemotherapy in children with lymphoma and explored the clinical application value of conventional echocardiography, serological makers, and ultrasound strain parameters to early detect the chemotherapy-induced cardiac dysfunction and to provide a new rationale for the clinical monitoring of the cardiotoxicity of ANTs.

## Materials and methods

### Study population

**Figure 1:**
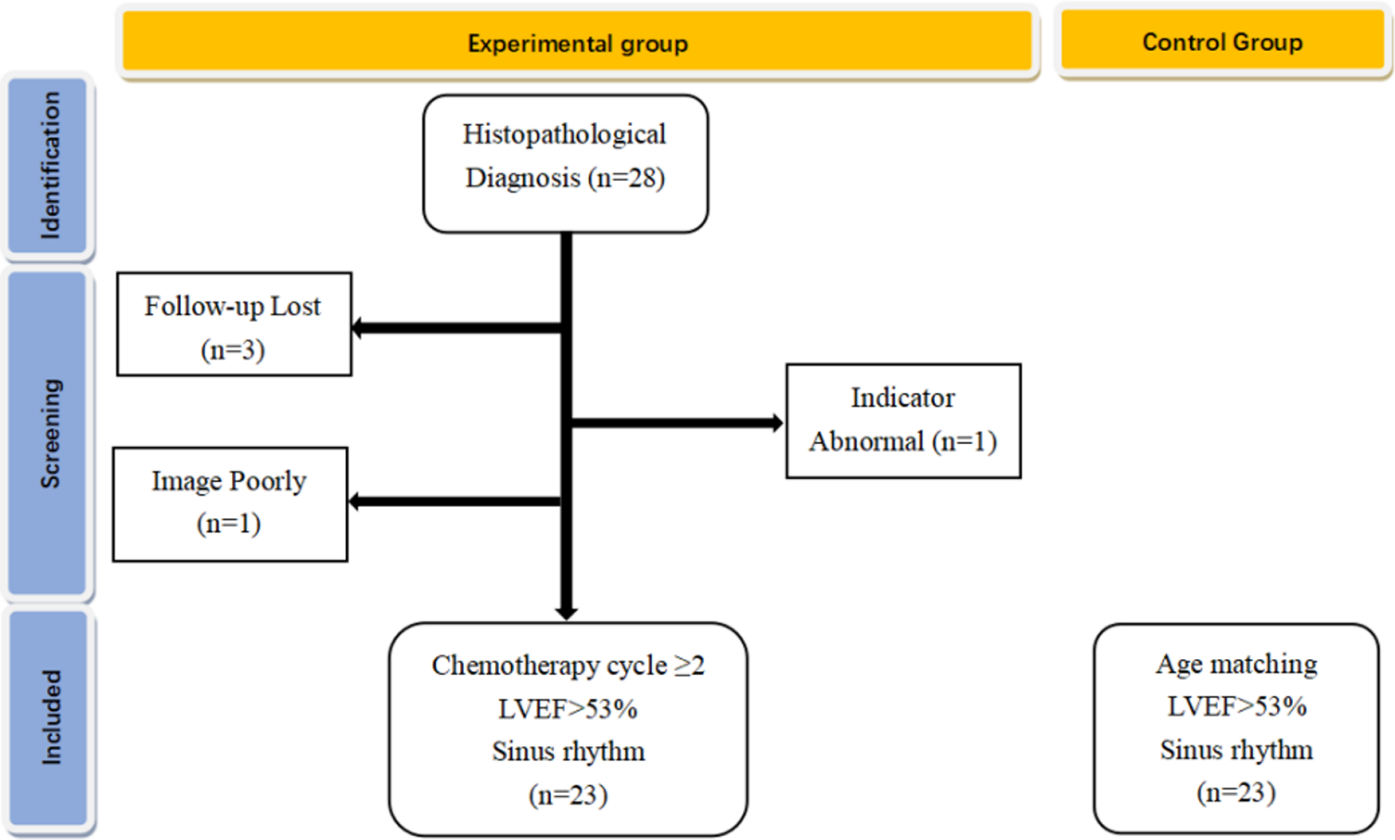
Flowchart of this cohort: LVEF, left ventricular ejection fraction.

From January 2019 to March 2020, 28 children with lymphoma-confirmed histopathological diagnosis were first selected at Children's Hospital of Chongqing Medical University, who completed at least two courses of chemotherapy with normal LVEF (>53%) and sinus rhythm. The exclusion criteria included congenital heart disease, myocarditis, severe arrhythmia, cardiac function impairment due to anemia and nephrotic syndrome, and poor image quality due to excessive obesity and severe pneumonia. Therefore, we excluded 5 patients (3 patients interrupted follow-up, 1 patient had abnormal cardiac and mechanical issues before chemotherapy, and 1 patient was excluded for poor image quality due to obesity), and a total of 23 subjects were included in the final analysis; the flowchart of this cohort shown in Figure 1. Twenty-three healthy children of similar age were recruited from pediatric clinics. This study was approved by the Ethics Committee of Children's Hospital of Chongqing Medical University of Public Health, Chongqing Medical University (2016-34). All participants provided their written informed consent.

### Clinical data and serologic marker

The age, height, weight, blood pressure, heart rate, and chemotherapy regimen of every child were recorded, and the child's body surface area and the cumulative amount of Adriamycin after every two courses of treatment were measured. Venous blood samples were taken from each kid before chemotherapy and within 24 h after every two chemotherapies to measure the serum levels of creatine kinase isoenzymes MB (CK-MB) and troponin I ultra (TnI-Ultra) before and after chemotherapy.

### Echocardiography and blood pressure

The same physician performed echocardiograms before chemotherapy and within 24 h after every two courses of chemotherapy using a GE system (E9, GE Healthcare, Norway) and M5S transducer (3.5 MHz) as per current guidelines (4, 14, 15). Post-processing was performed by a sonographer using GE EchoPAC 204. After the subjects rested in the left recumbent position and breathed calmly, electrocardiogram (ECG) leads were connected, and blood pressure was measured simultaneously. The main collected images included: M-mode echocardiography in the parasternal LV long-axis view and ring motion spectrum on the mitral annular side using tissue Doppler imaging (TDI). For strain parameters, two-chamber, three-chamber, and four-chamber views of LV with more than three cardiac cycles were obtained continuously from the apical long-axis view of the LV and stored in uncompressed format.

### Conventional echocardiography

The images were obtained in DICOM format, and the left atrial myocardial motion was depicted in the apical four-chamber view and apical two-chamber view with the matching software EchoPAC 204 to calculate the left atrial end-diastolic dimension (LAEDd), left atrial end-diastolic volume (LAEDV), left atrial end-systolic volume (LAESV), and the biplane left atrial ejection fraction (LAEF) and left atrial volume index (LAVI); in the mode of Auto-EF, the apical two-chamber and four-chamber end-systolic left ventricular intimal surfaces were depicted, and LVEF was calculated by the biplane Simpson method; the crest mitral valve orifice blood flow rate was measured by spectral Doppler in the apical four-chamber view to calculate E/A.

### Left ventricular strain

**Figure 2:**
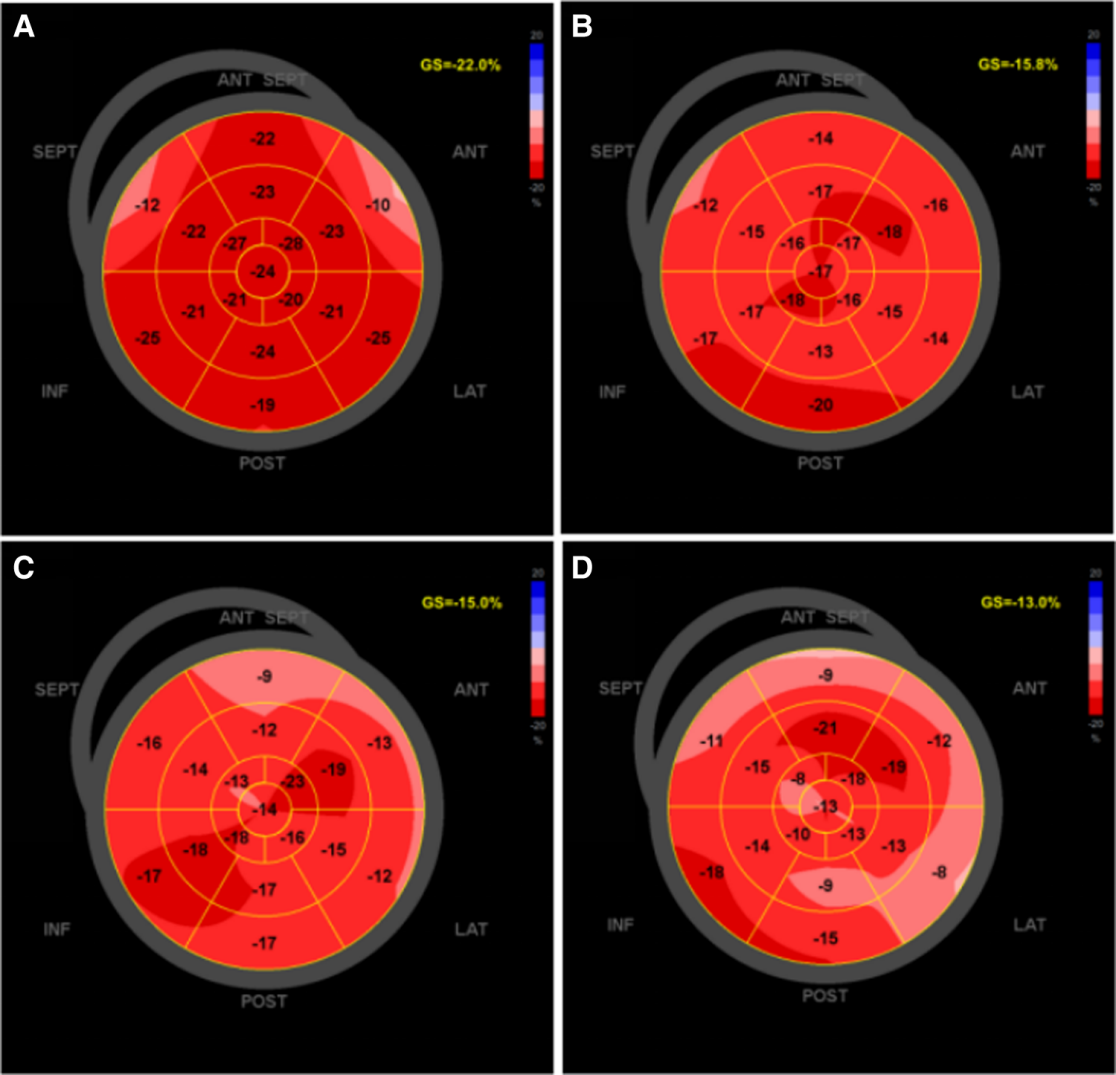
The software offline analysis schematic of left ventricular GLS in EchoPAC 204: light-colored areas indicate the decrease in myocardial longitudinal strain. (**A**) Bull's-eye of left ventricular GLS before chemotherapy; (**B**) after second cycle of chemotherapy; (**C**) after fourth cycle of chemotherapy; (**D**) after sixth cycle of chemotherapy. GLS, global longitudinal strain.

In auto function imaging (AFI), the corresponding standard view of three-chamber, four-chamber, and two-chamber heart (APLAX, 4CH, 2CH) was selected. The software can automatically track the LV membrane surface to form a range of interest through the electrocardiogram; manually depict or finetune if necessary; calculate the GLS of LV in APLAX, 4CH, and 2CH; and the software automatically calculates the average value GLPSAvg (LVGLS) (Figure 2). At the same time, the left ventricular layered analysis technique takes a left ventricular systolic subendocardial myocardium, medial myocardium, and epicardial myocardium global longitudinal strain; on the basis of obtaining the bovine ophthalmogram, the myocardial work parameters (including segments and overall) are calculated by EchoPAC 204 version (GE Healthcare) software: global work index (GWI), global constructive work (GCW), global wasted work (GWW), and global work efficiency (GWE).

### Left atrial strain

**Figure 3:**
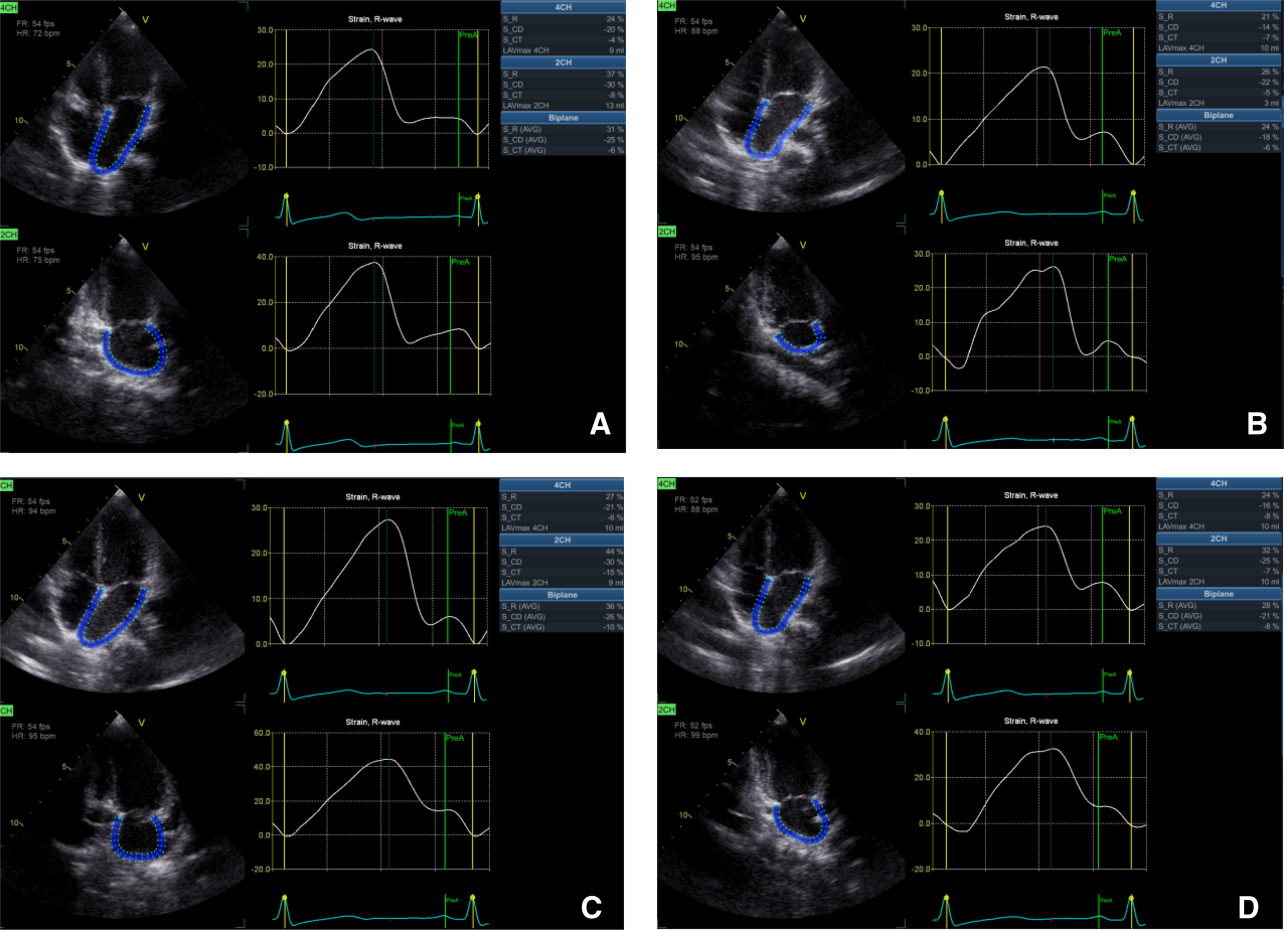
The software offline analysis schematic of left atrial longitudinal strain in EchoPAC 204: (**A**) left atrial longitudinal strain before chemotherapy; (**B**) after second cycle of chemotherapy; (**C**) after fourth cycle of chemotherapy; (**D**) after sixth cycle of chemotherapy.

The left atrial global strain was automatically tracked at a finishing time of diastole (the mitral valve was closed), left atrial strain of reservoir phase (LASr), left atrial strain of conduit phase (LAScd), left atrial strain of contraction phase (LASct) at 4CH and 2CH were calculated and record it respectively, and their mean values BiSr, BiScd, and BiSct, recorded as LASr, LAScd, LASct (Figure 3). All parameters were measured at least three times and averaged.

### Statistical analysis

Statistical analysis was performed using SPSS 25.0 software. LVGLS, LAScd, and Sct were negative values, and absolute values were taken for a description in this paper; normally distributed data and approximately normally distributed data are expressed as the mean ± standard deviation (SD); differences between groups were analyzed with independent-sample *t* test, Single factor variance analysis, and repeated measures analysis of variance (ANOVA); non-normally distributed data are expressed as the median ± interquartile range and between groups were analyzed with the Wilcoxon signed rank test. Correlation analysis was performed using Pearson correlation analysis, and diagnostic efficacy was assessed using binary logistic regression analysis and receiver operating characteristic (ROC) curve. *p* < 0.05 was considered statistically significant.

## Results

### Clinical information

The study population (*n* = 23) consisted of 20 males and 3 females with a mean age of 9.34 ± 3.59 years (range 5–15 years). They all completed at least two chemotherapy courses (cumulative ANT dose 50 mg/m^2^): 19 patients completed four chemotherapy courses (cumulative ANT dose 100 mg/m^2^), 12 patients completed six chemotherapy courses (cumulative ANT dose 150 mg/m^2^), and 8 patients completed eight chemotherapy courses (cumulative ANT dose 230 mg/m^2^). Twenty-three children developed varying degrees of nausea, vomiting, and myelosuppression after chemotherapy only within different chemotherapy cycles, which resolved spontaneously after the end of chemotherapy without other discomforts such as palpitations or dizziness and had no significant abnormalities in myocardial markers or electrocardiograms. In addition, only 1 of the 23 patients died of severe infection within 1 year after the end of chemotherapy. The control group consisted of 23 patients, 20 males and 3 females, with an average age of 9.42 ± 3.15 years (age range, 4–15 years). There were no obvious differences in age, weight, height, and gender distribution between the control and study groups (*p* > 0.05). The diastolic blood pressure (DBP) was significantly lower than healthy controls (*p* < 0.05). There was no statistical significance in body surface area (SA), heart rate (HR), and systolic blood pressure (SBP) between 23 children with lymphoma before chemotherapy and 2, 4, and 6–8 times after chemotherapy and the control group (*p* > 0.05) (Table 1). During the whole chemotherapy period, serum TnI-Ultra was within normal limits in children with lymphoma, and most of them were not detected in serum and rarely fluctuated, so monofactor analysis of variance displayed that TnI-Ultra changes were not statistically significant. CK-MB fluctuated more in the normal range than TnI-Ultra, but none of the changes were statistically significant (Table 2).

**Table 1:** Comparison of general clinical data and conventional ultrasound parameters of children with lymphoma and healthy control group.

Variables	Control group (*n* = 23)	Before chemotherapy (*n* = 23)	Chemotherapy cycle 2 (*n* = 23)	Chemotherapy cycle 4 (*n* = 19)	Chemotherapy cycle 6 (*n* = 12)	Chemotherapy cycle 8 (*n* = 8)
Weight (kg)	36 (19–47)	30 (20–38)	29 (20.5–40)	30.25 (22–40.13)	28 (22–37.25)	35 (26.5–53)
BSA (m^2^)	1.2 (0.77–1.4)	1.15 (0.8–1.25)	1.12 (0.82–1.3)	1.13 (0.87–1.28)	1.03 (0.87–1.2)	1.2 (1.03–1.55)
SBP (mmHg)	98.57 ± 5.88	100.83 ± 11.87	103.13 ± 12.94	97.37 ± 11.47	95.5 ± 9.35	101 ± 5.73
DBP (mmHg)	68 (61–80)	62 (60–70)*	62 (60–66)	62 (60–65.75)	61.5 (60–67.25)	63 (60–73)
HR	91.57 ± 8.88	97.22 ± 8.28	92.52 ± 6.76	97.74 ± 10.26	98.17 ± 9.05	89.83 ± 4.62
LVEF (%)	0.56 (0.55–0.60)	0.57 (0.55–0.59)	0.59 (0.55–0.61)^#^	0.55 (0.52–0.60)	0.56 (0.51–0.63)	0.57 (0.55–0.62)
E/A	1.57 (1.49–1.63)	1.48 (1.28–1.59)	1.46 (1.36–1.58)	1.51 (1.36–1.65)	1.51 (1.45–1.68)	1.56 (1.33–1.73)
LAEDd (mm)	4.41 ± 0.87	4.19 ± 0.55	4.37 ± 0.60	4.38 ± 0.56	4.46 ± 0.65	4.66 ± 0.46^#^
LAEDV (ml)	26 (17–33)	21.64 (17–25)	23 (18–28)	22 (15.25–28.5)	22 (17.25–30)	21 (19–29)
LAESV (ml)	7 (5–13)	6 (4–7)	5 (4–8)	8 (5–10)	6 (6–7.75)	7 (4–8)
LAEF (%)	0.67 ± 0.02	0.72 ± 0.01	0.72 ± 0.02	0.66 ± 0.01	0.69 ± 0.02	0.71 ± 0.03
LAVI	15.99 ± 2.79	13.36 ± 5.05*	14.22 ± 1.11	14.08 ± 1.47	17.15 ± 1.91	15.77 ± 2.09

BSA, body surface area; SBP, systolic blood pressure; DBP, diastolic blood pressure; HR, heart rate; LVEF, left ventricular ejection fraction; E/A, maximum flow rate in early mitral relaxation/left atrial systolic maximum flow rate; LAEDd, left atrial end-diastolic dimension; LAEDV, left atrial end-diastolic volume; LASEV, left atrial end-systolic volume; LAEF, left atrial ejection fraction; LAVI, left atrial volume index.

Data are expressed as mean ± SD or median ± interquartile range.

* *p* < 0.05 compared with controls.

^#^ *p* < 0.05 compared the last chemotherapy group.

**Table 2:** Comparison of myocardial markers in children with lymphoma before and after chemotherapy.

	Before chemotherapy (*n* = 23)	Chemotherapy cycle 2 (*n* = 23)	Chemotherapy cycle 4 (*n* = 19)	Chemotherapy cycle 6, 8 (*n* = 20)	*F*	*p*
CK-MB	1.12 ± 1.56	0.65 ± 0.41	0.82 ± 0.38	0.83 ± 0.36	1.38	1.000
TnI-Ultra	0.003 ± 0.13	0.000 ± 0.00	0.006 ± 0.01	0.006 ± 0.01	1.20	0.340

CK-MB, creatine kinase isoenzymes MB; TnI-Ultra, troponin I ultra.

### Cardiac function assessment

LVEF, E/A, LAEDd, LAEDV, LAESV, and LAVI determined by the Simpson method were in the normal range, and there was no statistical significance between before and after chemotherapy with the control group (*p* > 0.05). After chemotherapy, the data of LVEF determined by the Simpson method decreased in the normal range compared with that before chemotherapy (*p* > 0.05); LAVI before chemotherapy in children with lymphoma were lower when compared with the normal group (15.99 ± 2.79 vs. 13.36 ± 5.05, *p* < 0.05) (Table 1).

**Figure 4:**
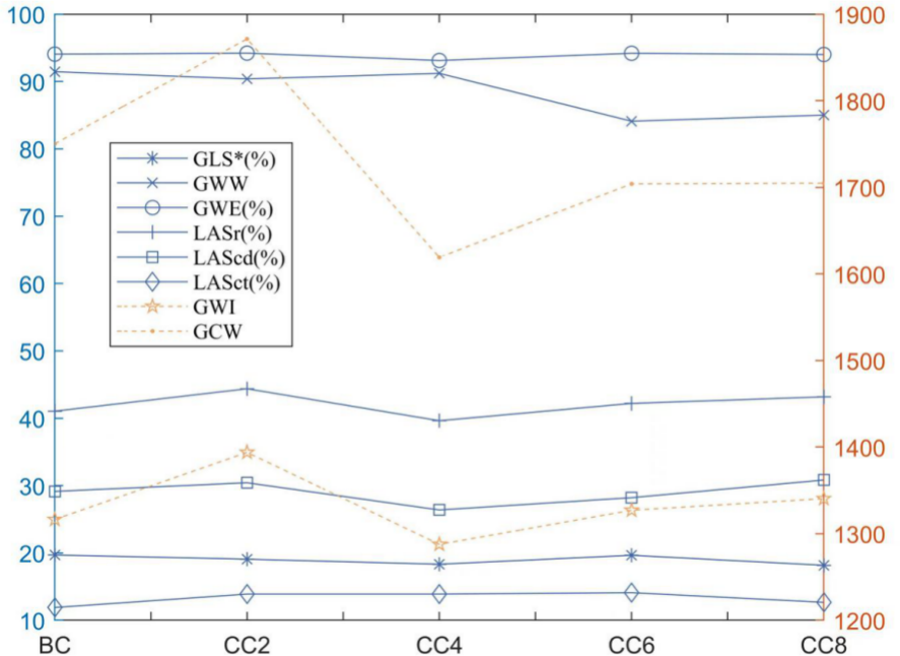
Line plot of strain data in the children with lymphoma before and after chemotherapy: *p < 0.05 compared with before chemotherapy. BC, before chemotherapy; CC2, after second cycle of chemotherapy; CC4, after fourth cycle of chemotherapy; CC6, after sixth cycle of chemotherapy; CC8, after eighth cycle of chemotherapy.

LVGLS, GWI, GWW, and GWE were significantly lower than healthy controls (*p* < 0.05), while GCW, LASr, LAScd, and LASct were not significantly different between the two groups (*p* > 0.05). Compared with that before chemotherapy, the mean values of GLS in children with lymphoma after chemotherapy at cycles 2, 4, 6, and 8 showed a decreasing trend at cycles 2 and 4 [19.39 (17.63–20.96) vs. 17.55 (16.94–20.59), *p* < 0.05]. However, they fluctuated at cycles 6 and 8 (*p* > 0.05) (Table 3, Figure 4). There was no significant difference between GWI, GCW, GWW, GWW, and GWE treatment (*p* > 0.05).

**Table 3:** Comparison of strain parameters of children with lymphoma and healthy control group.

Variables	Control group (*n* = 23)	Before chemotherapy (*n* = 23)	Chemotherapy cycle 2 (*n* = 23)	Chemotherapy cycle 4 (*n* = 19)	Chemotherapy cycle 6 (*n* = 12)	Chemotherapy cycle 8 (*n* = 8)
GLS (%)	21.8 (20.21–23.6)	18.93 (18.121.1)*	19.39 (17.63–20.96)	17.55 (16.94–20.59)**	19.35 (17.04–21.22)	18.86 (16.25–20.18)
GWI	1,580.87 ± 189.99	1,316.36 ± 230.13*	1,393.90 ± 269.90	1,287.68 ± 355.53	1,327.00 ± 259.01	1,340.43 ± 162.39
GCW	1,869.00 ± 154.62	1,750.27 ± 284.52	1,871.29 ± 297.99	1,619.05 ± 385.33	1,703.91 ± 269.74	1,704.71 ± 226.45
GWW	54 (33–79)	82 (59–103)*	88 (73–102)	86 (56.5–140.75)	88 (55–107.5)	66 (49–114)
GWE (%)	97 (96–98)	94 (92–96)*	95 (94–96)	93.5 (91.25–95.75)	94.5 (92.25–95.75)	94 (93–96)
LASr (%)	41 (38–46)	41 (37–45)	44 (37–50)	39.5 (34–44.5)	37.5 (30.5–50.25)	41 (38–44)
LAScd (%)	29 (28–35)	30 (24–32)	30.45 (26–35)	26 (22–31)	25.5 (22–34.5)	30 (28–36)
LASct (%)	10 (9–12)	12 (9–14)	13.85 (11–18)	12.5(10–15.5)	14(8.5–15)	12(12–15)

GLS, global longitudinal strain; GWI, global work index; GCW, global constructive work; GWW, global wasted work; GWE, global work efficiency; LASr, left atrial strain of reservoir phase; LAScd, left atrial strain of conduit phase; LASct, left atrial strain of contraction phase.

* *p* < 0.05 compared control group.

** *p* < 0.05 compared the last chemotherapy group.

Among them, the monofactor analysis of variance test was used to compare the degree of LS mean to reduce in LV systolic subendocardial myocardium, medial myocardium, and subepicardial myocardium after chemotherapy in comparison to that before chemotherapy to obtain the outcome: left ventricular myocardial LS revealed a regular pattern of epicardial layer < middle layer < subendocardial layer (*p* < 0.05) whether before chemotherapy or after chemotherapy; and the degree of decreased also showed revealed a regular pattern of epicardial layer < middle layer < subendocardial layer while the difference was not significant (*p* > 0.05) (Table 4).

**Table 4:** Comparison of LS in subendocardial, middle, and subepicardial layers of children with lymphoma before and after chemotherapy.

Variables	Before chemotherapy (*n* = 23)	After chemotherapy (*n* = 23)	Diversification (*n* = 23)
Subendocardial	24.44 ± 4.93*	21.09 ± 3.53*	2.89 ± 5.44
Mid-myocardial	21.13 ± 4.16*	18.30 ± 2.93*	2.45 ± 4.55
Subepicardial	18.27 ± 3.67*	15.93 ± 2.67*	1.93 ± 3.96

* *p* < 0.05 compared with each layer.

When compared to the control group, lymphoma children's LASr, LAScd, and LASct were in the normal range; about 14.11% (12 of 85 cases) had LASr lower than 35%. LASr, LAScd, and LASct were slightly increased after the second cycle of chemotherapy but decreased after the fourth cycle of chemotherapy, and the overall mean value was below that of the healthy control group, and slightly increased again in the sixth and eighth cycles after chemotherapy. The overall mean value exceeded that before chemotherapy but was lower than that in the second cycle of chemotherapy (Figure 4); at the same time, there was no significant difference in comparison to the two groups (*p* > 0.05). Correlation analysis showed that LASr was weak positively correlated with LAVI, LVEF, GLS, and LASct (*r* = 0.486–0.565, *p* < 0.01); LAScd was weak positively correlated with E/A, LAEF, and LAVI (*r* = 0.306–0.486, *p* < 0.01), moderate positively correlated with LVEF and GLS (*r* = 0.506–0.529, *p* < 0.01), and strong positively correlation with LASr (r = 0.851, *p* < 0.01); LASct was weak positively correlated with LAVI (*r* = 0.391, *p* < 0.01), moderate positively correlated with LASr, and there was no significant association with E/A, LAEF, LVEF, GLS, and LAScd (*r* = −0.138 to 0.229) (Table 5).

### Prediction CTRCD by STE

**Figure 5:**
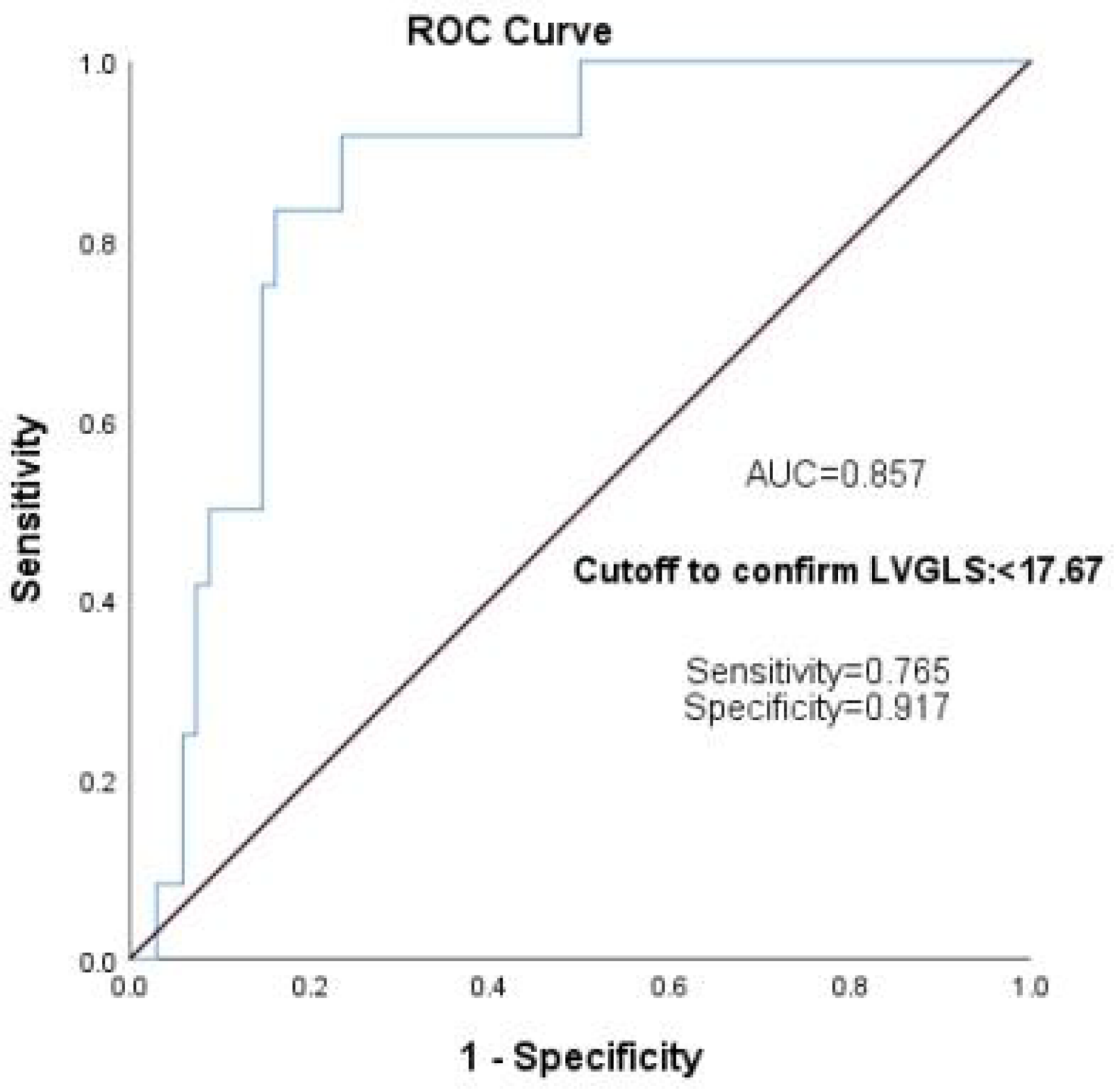
GLS predicts the ROC curve of CTRCD in the children with lymphoma after chemotherapy. GLS, global longitudinal strain; ROC, receiver operating characteristic; CTRCD, cancer treatment-related cardiac dysfunction.

According to the diagnostic criteria for CTRCD (4), LVEF decreased by more than 10% after chemotherapy in comparison to that before chemotherapy or cardiac abnormality-related symptoms with LVEF decreased by more than 5%. In this research, 13 of 85 cases met the diagnostic criteria for asymptomatic CTRCD, with an incidence of about 15.29%; all strain data using one-way ANOVA showed that GLS, GWI, GCW, LASr, and Scd were closely associated with CTRCD (Table 6); further multivariate logistic regression analysis was used to exclude GWI, GCW, LASr, and Scd, and showed that GLS was the most sensitive predictor for detecting patients at lofty risk of anthracycline-related cardiotoxicity [regression coefficient *B* = −0.965, Exp (*B*) = 0.381, *p* < 0.05]. When the absolute value of LVGLS was < 17.67, the sensitivity for predicting CTRCD was 0.765, the specificity was 0.917, and the area under the curve (AUC) = 0.857 (Figure 5).

**Table 5:** The Pearson correlation of LA parameters and GLS of children with lymphoma.

r	E/A	LAVI	LAEF	LVEF	GLS	LASr	LAScd	LASct
E/A	1	** **	** **	** **	** **	** **	** **	** **
LAVI	0.373**	1	** **	** **	** **	** **	** **	** **
LAEF	0.059	0.136	1	** **	** **	** **	** **	** **
LVEF	0.135	0.327**	0.183	1	** **	** **	** **	** **
GLS	0.066	0.393**	0.248*	0.568**	1	** **	** **	** **
LASr	0.228*	0.565**	0.295**	0.513**	0.486**	1	** **	** **
LAScd	0.373**	0.486**	0.306**	0.506**	0.529**	0.851**	1	** **
LASct	−0.138	0.391**	0.07	0.229*	0.145	0.608**	0.179	1

E/A, maximum flow rate in early mitral relaxation/left atrial systolic maximum flow rate; LAVI, left atrial volume index; LAEF, left atrial ejection fraction; LVEF, left ventricular ejection fraction; GLS, global longitudinal strain; LASr, left atrial strain of reservoir phase; LAScd, left atrial strain of conduit phase; LASct, left atrial strain of contraction phase.

The correlation size is expressed in *r*: *r* = 0.3–0.5 for weak correlation, *r* = 0.5–0.7 moderate correlation, *r* = 0.7–0.9 strong correlation; −, negative correlation.

* At 0.05 level (double tail), significant correlation.

** At 0.01 level (double tail), significant correlation.

**Table 6:** Summary of the association between strain data and CTRCD.

Variables	Mean ± SD	*F*	*p*
GLS	19.04 ± 2.48	13.21	<0.01
GWI	1,310.83 ± 271.12	4.88	0.03
GCW	1,716.45 ± 308.44	6.11	0.02
GWW	89.91 ± 42.41	0.587	0.45
GWE	93.83 ± 2.87	1.42	0.24
Subendocardial	23.67 ± 4.51	1.03	0.31
Mid-myocardial	20.61 ± 4.00	1.93	0.17
Subepicardial	18.00 ± 3.70	2.66	0.11
LASr	41.30 ± 8.76	6.01	0.02
LAScd	28.44 ± 6.83	4.32	0.04
LASct	12.87 ± 4.86	1.76	0.19

GLS, global longitudinal strain; GWI, global work index; GCW, global constructive work; GWW, global wasted work; GWE, global work efficiency; LASr, left atrial strain of reservoir phase; LAScd, left atrial strain of conduit phase; LASct, left atrial strain of contraction phase.

*p* < 0.05, showed a strong association with CTRCD.

## Discussion

The outcome displayed that LVEF, E/A, LAEDd, LAEDV, LAESV, and LAVI measured by the Simpson method were within the normal range, and there was no statistical significance before and after chemotherapy. After chemotherapy, some children showed an obvious decrease in LVEF by the biplane Simpson method, suggesting abnormal left ventricular function after chemotherapy and reaching the CTRCD diagnostic criteria, but the difference among the chemotherapy groups were not statistically significant. Cardiac reserve makes changes in LVEF less than 10% not necessarily representative of actual changes in the systolic function (16), and changes in LVEF often become apparent years after receiving the associated influence, particularly in pediatric cancer (4), which limits the validity of biplane Simpson LVEF subsequent to our study subjects. However, studies (7) have proposed that three-dimensional spot tracking echocardiography (3D-STE) allows the quantification of myocardial deformation in three spatial dimensions, overcoming the inherent limitations of 2D LVEF, which mainly depends on load conditions and geometric assumptions, to accurately assess LV function and detect subclinical cardiotoxicity.

The left atrial changes are asymmetric and difficult to measure quantitatively, and the left atrial size is influenced by many factors, which leads to unreliable results such as LAESV. We discovered that LAVI before chemotherapy in lymphoma children was clearly lower than those in the control group, LAVI predicts diastolic dysfunction by reflecting the magnitude of LV filling pressure, and monitors LV diastolic function more accurately than E/A and LAD (17). Following 195 individuals with acute cardiac failure, Almeida et al. (18) discovered that the optimum cutoff value for heart disease with intact ejection fraction was 34 ml/m^2^. Therefore, when 34 ml/m^2^ was used as the boundary, LAVI was in the normal range in all children (only two cases exceeded 29 ml/m^2^), but the minimum age of this study was 50 years, which was different from our study subjects in age. Unfortunately, we lacked the criteria for the diagnosis of LAVI abnormalities in children, so the predictive value of LAVI in children may be underestimated.

CK-MB and TnI-Ultra are widely used as early clinical indicators of myocardial injury. Compared with conventional troponins, high-sensitivity troponin has a much-reduced detection limit for detecting cTn as low as 1.90 or 1.20 pg/ml, allowing the early detection of minor myocardial damage (19). CK-MB and TnI-Ultra did not change apparently after chemotherapy in our research, possibly because troponin was inconsistently released through blood or it may also be due to some self-healing of ticker growth in children; it is unclear how serum markers should be applied for risk stratification of CTRCD importantly (20). In one study, cumulative anthracycline doses reached 0, 120, 240, and 360 mg/m^2^ in breast cancer patients, and serum Hs-cTnT concentrations after anthracycline chemotherapy showed a gradually increasing trend over four chemotherapy stages, while only one of our children had anthracycline concentrations reaching 350 mg/m^2^, mostly less than 100 mg/m^2^. This could also explain why serum indicators in our investigation did not indicate any changes (21). In addition, the diverse results of serological myocardial markers in exploring early cardiac function impairment caused by chemotherapy (21, 22) may be related to chemotherapeutic drugs and interindividual differences.

Compared with conventional ultrasound data and myocardial markers, LS may be abnormal in the early stage, and it directly reflects myocardial function rather than cardiac pump function. STE quantitatively analyzes myocardial motion by speckle tracking, and LS reflects the function of longitudinal myofibers under the endocardium; anthracycline chemotherapeutic drugs can cause uneven degeneration and fibrosis of the left ventricular myocardium, such as the cardiac histopathological findings of doxorubicin intoxication are characterized by vacuolar degeneration of muscle cells (23–25), because about 70% of the LV is composed of longitudinal muscles, and the endocardial layer is responsible for longitudinal contraction. The endocardium is the area most prone to hypoperfusion and ischemia, so anthracycline cardiotoxicity is often characterized by a decrease in endocardial longitudinal function in the early stage (26). In our study, the LS values of each layer of the myocardium in each segment of the LV in children maintained a certain gradient, that is, the absolute LS values of each layer of the LV myocardium decreased layer by layer from the endocardium to the epicardium, suggesting that the longitudinal deformation of the myocardium has regular transmural heterogeneity. With the accumulation of anthracycline dose causing myocardial damage or acute cardiotoxicity, the longitudinal strain of each layer of myocardium decreased. The degree of decrease also revealed a regular pattern of epicardial layer < middle layer < subendocardial layer, which is in accord with the results of Kang et al. that the endocardium is more sensitive and more susceptible to damage, but it lacks statistical significance to draw further conclusions.

In this research, we discovered that GLS and myocardial work parameters in children were significantly lower than controls before chemotherapy, which may indicate that cardiac muscle strength was impaired in lymphoma children before chemotherapy; it may be related to the role of inflammatory mediators in the process of myocardial remodeling in LV and myocardial infiltration of cancer cells in lymphoma patients. This result is consistent with the study of Akam-Venkata et al. (27). After chemotherapy, LVGLS remained progressively decreasing, nadir at cycle 4 after chemotherapy; at this time, the cumulative dosage of the child is about 100 mg/m^2^. Another study (28) compared the cardiac function of 86 young and middle-aged patients after ANTH chemotherapy, and it pointed out that LVGLS could predict cardiotoxicity when ANTH reached 150 mg/m^2^, indicating that the cardiotoxicity of ANTH in children was greater than that of young and middle-aged patients. The data of cycles 6 and 8 are volatile, which may indicate the self-healing of the heart function in the children themselves, or it may be because the study data are small and not representative. LVGLS had the ability to diagnose CTRCD and its changes occurred earlier than those of other conventional ultrasound parameters and myocardial markers, so LVGLS is one of the sensitive indicators for evaluating whole left ventricular myocardial function (26). LVGLS can be used as an indicator to evaluate myocardial function in patients during chemotherapy, detect early cardiac dysfunction, predict the occurrence of CTRCD, and guide cardioprotective therapy (2, 6, 29–32).

One study (33) pointed out that GLS did not show a relationship with LV contractility, reflecting ventriculoatrial coupling (VAC). GWI showed a stronger linear relationship with LV inotropy compared to GLS (34). In our study, GLS trendily decreased after chemotherapy, while the myocardial work parameters did not change significantly before and after chemotherapy. Based on the predictive effect of GLS on CTRCD, we proposed this speculation that hemodynamic changes precede inotropic impairment in the effects of anthracyclines on the heart. In addition, our results showed that GWI and GCW were closely related to CTRCD (35), but they were excluded in the multivariate logistic regression analysis, and more meaningful analysis results might have been obtained if the sample size had been expanded. However, the prediction of CTRCD is definitely not a significance of myocardial work. If it detects early hemodynamic disturbances, early intervention can be performed to avoid myocardial injury.

Abnormal LA function is also caused by myocardial fibrosis leading to abnormal myocardial movement. Therefore, LA strain could monitor the longitudinal, radial, and circumferential difference of LA myocardial function by STE, but the left atrial myocardial fibers are thin. At present, it is advocated that strain measurement of the left atrium be done as a whole, without separating the levels and parts (36). It has been shown that the value of LA strain is decreased in LVDD, LV end-diastolic pressure is LA afterload, and LA function can reflect LV function to some extent (37). We focused on the GLS of the left atrium in the four-chamber view and the two-chamber view, namely, LASr, LAScd, LASct, and their mean value. LASr corresponds to left atrium early diastole, persists from mitral valve closure to mitral valve opening, is the maximum relaxation strain value of left atrium wall, is closely related to LVDD, and less than 35% can be considered LVDD. In the study, 12 out of 85 cases had LASr lower than 35% when E/A and LAVI were in the normal range, indicating that LVDD caused by abnormal left atrial function may occur after chemotherapy. LAScd corresponds to LA mid-diastole and LA emptying, persists from mitral valve opening to LA contraction, is associated with LA compliance and LV diastolic function, and is prone to early changes. LASct corresponds to left atrial systolic strain, persists from LA contraction to mitral valve closure, and reflects LA systolic ability (38, 39). The results showed that there was no obvious difference in LASr, LAScd, and Sct before chemotherapy between children with lymphoma and healthy controls, that is, left atrial strain indexes were comparable; LASr, LAScd, and LASct after chemotherapy had some fluctuation suggesting that LASr and LAScd changes in left atrial strain parameters changed earlier than conventional parameters such as E/A and LAVI; however, the difference among the different groups was not statistically significant. Thus, no clear changes or trends in LASr, LAScd, and LASct were discovered in all children after ANTH chemotherapy in this experiment, which may be due to the stronger compensatory ability of the children's heart and the smaller cumulative dose of ANTH in all observed subjects (40).

Some scholars have proposed that left atrium peak strain can be used to detect left ventricular filling pressure (41). The result showed that LASr and LAScd were closely associated with CTRCD; however, no predictive value of left atrial strain value in the diagnosis of CTRCD was analyzed by logistic regression. That is, our study failed to predict left ventricular function changes after chemotherapy in children with lymphoma by left atrial strain in response to left ventricular filling pressure. Further correlation analysis showed that LASr and LAScd were moderate positively correlated with LVEF and GLS. LASct was only positively correlated with LAVI but not significantly correlated with LVEF and GLS. LVEF and GLS are currently the main indicators of ultrasound for the evaluation of LV function. This suggests that we can use the left atrial strain to monitor the myocardial toxicity and LASr and LAScd may detect myocardial dysfunction earlier than LASct, which is alike to the results of other studies (42). It may be that left ventricular lesion and relaxation reduce atrial catheter function (LAScd) and left atrial stiffness, that is, left atrial maximum relaxation, resulting in decreased LASr; in order to ensure left ventricular blood flow, LA contraction (LASct) is enhanced as a compensatory mechanism in the early stages, but LASct also decreases due to LA dilatation and stiffness as dysfunction progressed.

In addition to 2D-SDE, 3D-derived spot tracking as a reproducible ultrasound technique also clearly demonstrates changes in LV volume, mass, and function (43), and studies (44) indicate that 3D-STE has lower time variability in cancer patients undergoing chemotherapy with stable left D function compared to 2D estimates. However, the temporal resolution and image quality may limit the feasibility of 3D-STE (45). Using 3D-STE to study the myocardial function after chemotherapy in children with lymphoma may help us to deeply understand the myocardial function changes in children, and more simply detect the early changes, in order to assist in clinical early detection and early treatment.

### Limitations

There are some limitations to this study. The sample size is small, and we did not perform long-term follow-ups of children with lymphoma following chemotherapy and could not determine the trends in GLS following discontinuation of ANT, but we mainly explored the early changes. The expert consensus document of the American Society for Echocardiography (ASE) and the European Association of Cardiovascular Imaging (EACVI) recommended LVEF criteria were not met, but a GLS decrease of >15% from baseline can be considered subclinical CTRCD (4, 46, 47). We did not routinely assess the cardiac function by STE, which caused strain to vary in different age groups. E/A and LAVI were in the normal reference range in all cases in the observation group and merely LASr was abnormal; the relationship between LASr, Scd, and Sct with conventional ultrasound parameters to assess LVDD could not be further analyzed.

## Conclusion

LVGLS is a more sensitive and earlier indicator to predict CTRCD compared with conventional echocardiography-related parameters and serological markers. GLS of each myocardial layer showed a certain regularity. Left atrial strain can be used for early monitoring of cardiotoxicity in children with lymphoma after chemotherapy.

## Data Availability

The raw data supporting the conclusions of this article will be made available by the authors, without undue reservation.
